# Dual-Specificity Phosphatases in Regulation of Tumor-Associated Macrophage Activity

**DOI:** 10.3390/ijms242417542

**Published:** 2023-12-16

**Authors:** Marina R. Patysheva, Elizaveta A. Prostakishina, Arina A. Budnitskaya, Olga D. Bragina, Julia G. Kzhyshkowska

**Affiliations:** 1 Laboratory of Translational Cellular and Molecular Biomedicine, National Research Tomsk State University, 634050 Tomsk, Russia; patysheva_mr@onco.tnimc.ru (M.R.P.); elprostakishina@yandex.ru (E.A.P.); arina.budnitskaya@gmail.com (A.A.B.); 2 Laboratory of Cancer Progression Biology, Cancer Research Institute, Tomsk National Research Medical Center, Russian Academy of Sciences, 634009 Tomsk, Russia; rungis@mail.ru; 3 Laboratory of Genetic Technologies, Siberian State Medical University, 634050 Tomsk, Russia; 4 Institute of Transfusion Medicine and Immunology, Medical Faculty Mannheim, Mannheim Institute of Innate Immunosciences (MI3), University of Heidelberg, 68167 Mannheim, Germany; 5 German Red Cross Blood Service Baden-Württemberg—Hessen, 69117 Mannheim, Germany

**Keywords:** tumor-associated macrophages, dual-specify protein phosphatase, mitogen-activated kinase, solid tumors

## Abstract

The regulation of protein kinases by dephosphorylation is a key mechanism that defines the activity of immune cells. A balanced process of the phosphorylation/dephosphorylation of key protein kinases by dual-specificity phosphatases is required for the realization of the antitumor immune response. The family of dual-specificity phosphatases is represented by several isoforms found in both resting and activated macrophages. The main substrate of dual-specificity phosphatases are three components of mitogen-activated kinase signaling cascades: the extracellular signal-regulated kinase ERK1/2, p38, and Janus kinase family. The results of the study of model tumor-associated macrophages supported the assumption of the crucial role of dual-specificity phosphatases in the formation and determination of the outcome of the immune response against tumor cells through the selective suppression of mitogen-activated kinase signaling cascades. Since mitogen-activated kinases mostly activate the production of pro-inflammatory mediators and the antitumor function of macrophages, the excess activity of dual-specificity phosphatases suppresses the ability of tumor-associated macrophages to activate the antitumor immune response. Nowadays, the fundamental research in tumor immunology is focused on the search for novel molecular targets to activate the antitumor immune response. However, to date, dual-specificity phosphatases received limited discussion as key targets of the immune system to activate the antitumor immune response. This review discusses the importance of dual-specificity phosphatases as key regulators of the tumor-associated macrophage function.

## 1. Introduction

Tumor-associated macrophages (TAMs) have key functions in the tumor microenvironment [1,2,3,4]. In the process, they are able to exhibit the activities of cells of innate immunity, such as the uptake of foreign particles by phagocytosis or endocytosis [1,5]. At the same time, macrophages are able to demonstrate antigen presentation and the production of soluble regulatory components of adaptive immunity cells [1,5]. Thus, macrophages, and TAMs in the context of the tumor, ensure the functioning of both the innate and adaptive immune response.

The ability of the tumor to polarize the immune cells in a way to support tumor growth is one of the mechanisms ensuring the development and progression of malignant neoplasms [6]. Thus, in the majority of ex vivo models, TAMs have the ability to support the tumor cells [2,5,7]. Moreover, clinical data demonstrate that TAMs’ density correlates with a poor prognosis for solid cancers [8]. In addition, experimental and clinical data suggest that TAMs have the ability to inhibit tumor chemo- and radio-resistance [9,10,11]. Thus, TAMs contribute overwhelmingly to tumor progression and contribute to the resistance to conventional cancer therapies. To date, several approaches have proved effective in tumor immunotherapy. Unfortunately, even such effective approaches as the use of checkpoint inhibitors for cytotoxic T-lymphocyte-associated protein 4 (CTLA-4) and programmed death-ligand 1 (PD-L1), as well as chimeric antigen receptor T-cells (CAR-T), have limitations and are still not fully successful. The regulation of TAM activity is considered to be a novel immunotherapeutic strategy in malignant neoplasms [12,13]. It is important to take into account that TAM regulation occurs at several levels, from the genomic to protein levels. TAM programming is possible both at the level of gene expression and signal transduction.

The regulation of macrophage activity takes place due to the synchronized action of protein kinases and phosphatases [14,15,16]. Protein kinases modify proteins by the phosphorylation of residues of amino acids containing hydroxyl groups or histidine groups. The main phosphorylation targets, serine, threonine, and tyrosine, account for 86.4%, 11.8%, and 1.8%, respectively, of the 6600 phosphorylation sites on 2244 human proteins [17]. In the regulation of protein kinase activity, dual-specificity tyrosine phosphatases are central due to their ability to dephosphorylate not only tyrosine sites but also serine/threonine sites [15]. Resting myeloid cells express a spectrum of protein kinases required to maintain cell viability. However, of especial interest is the change in the activity profile of protein kinases and phosphatases in the stimulus–response interaction or intracellular signal transduction. It should be noted that the intracellular localization of the protein kinase/phosphatase cascade remains an important aspect. While the study of macrophage protein kinases has previously received attention, the family of dual-specificity phosphatases remains poorly understood and requires further investigation [14,18,19].

A relevant task today is to identify key regulators of TAMs that equip them with antitumor activity. There is now compelling evidence for the essential role of DUSPs in macrophage activation. We hypothesize that DUSPs may be decision-making factors for the development of new immunotherapeutic anti-cancer drugs. In this review, we summarize the state-of-the art knowledge about the mechanisms of DUSP function in macrophages and discuss the results of the studies which addressed the role of DUSPs in malignancies.

## 2. Tumor-Associated Macrophages and Its Precursors in Tumor Immunity

**Figure 1:**
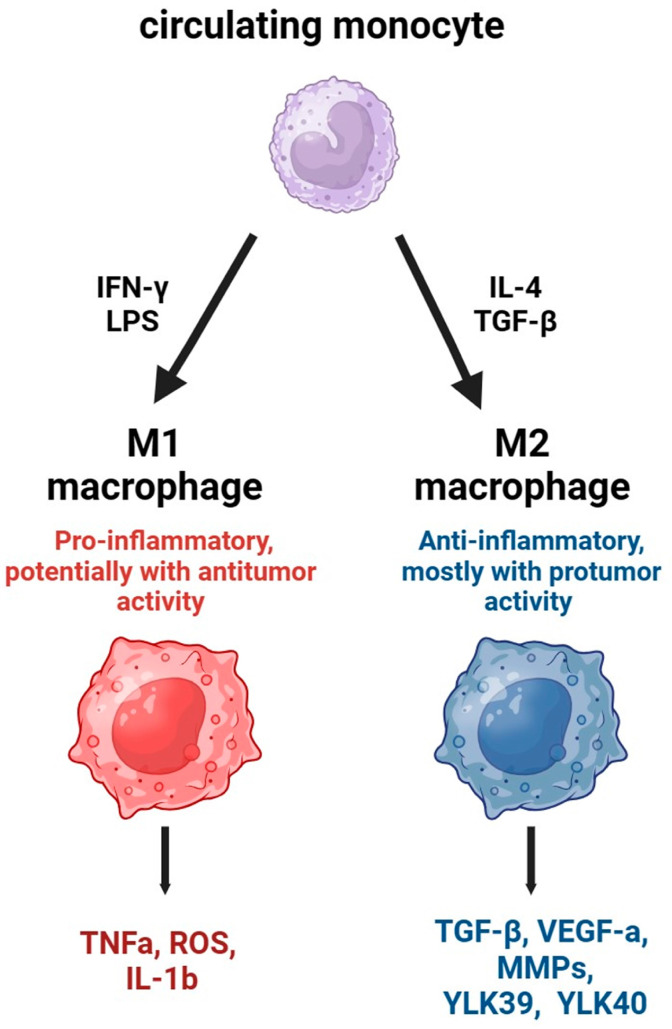
Major widely accepted vectors of macrophage polarization. LPS, IFN-y—prototype stimuli for polarization in M1 direction; IL-4, TGF-β—prototype stimuli for polarization in M2 direction. Selected M1- and M2-produced pro- and anti-inflammatory mediators related to tumor progression are listed.

Macrophages and their precursor monocytes are a front line of innate protection against pathogens and play an important role in the immunoregulation and maintenance of tissue integrity [20,21]. In the pathogenesis of malignancies, TAMs play a role as critical regulators that contribute to the tumor emergence, development, and response to current therapies [2,3,22]. The tissue macrophage population is characterized by heterogeneity, which is determined by macrophage ontogeny, the totality of regulatory stimuli received, and the location in a particular tumor compartment [3,23]. Depending on these factors, two opposite directions of the functional state of TAMs are possible—M1- or M2-like macrophages (Figure 1).

While the M1-like subtype of TAMs possesses a spectrum of antitumor activities, the M2-like population conversely supports tumor growth [2,24]. It has been experimentally shown that the M1-type is induced by interferon-γ (IFN-γ), bacterial membrane lipopolysaccharide (LPS), and a set of chemokines such as CXCL4, while differentiation into the M2-type is induced by interleukin 4, 10, and 13 (IL-4, IL-10, and IL-13), transforming growth factor β (TGF-β), and glucocorticoids, particularly dexamethasone [21,25,26] (Figure 1). M1 macrophages from cancer patients are characterized by high levels of reactive oxygen species (ROS) and release higher tumor necrosis factor-alpha (TNF-α) and express more interleukin 1 beta (IL-1β) [21,25,26], while M2 macrophages produce TGF-β, vascular endothelial growth factors α (VEGF-α), and metalloproteinases (MMPs) [21,25,26] (Figure 1). It should be noted that the differentiation of macrophages into M1- or M2-type is reversible. Thus, the introduction of appropriate inducers can change the biological profile of macrophages from M2 to M1, or vice versa [27]. However, using this simple distinction is not sufficient to define sets of TAMs populations. The current classification based on the study of single cells in the tumor microenvironment suggests seven types of TAMs, including interferon-stimulated, immunoregulatory, inflammatory-cytokine-enriched, lipid-associated, pro-angiogenic, resident tissue macrophage-like, and proliferating TAMs [28]. Based on clinical and laboratory data, it can be argued that, in many tumors, subtypes that support tumor growth are predominant among TAMs [2,8,24]. According to clinical observations, the presence of CD163, CD206, CD204, Stabilin1, or chitinase-like protein YLK39 and YLK40 markers in tumors, alone or in combination with the major macrophage marker CD68, positively correlates with a poor prognosis in solid tumors of the breast, colon and rectum, lung, ovary, and prostate [29,30,31,32,33,34,35].

It is now known that the pool of tissue macrophages is composed of resident macrophages originating from the embryonic yolk sac and embryonic liver and cells originating from circulating monocytes [36,37]. In malignant neoplasms, blood monocytes appear to be involved in the process of replenishing the TAM population. Monocytes are cells originating from the myeloid progenitor of hematopoiesis in the bone marrow, and subsequently exiting into the peripheral blood and migrating to tissues. Monocytes recruited into the blood are considered immature immune cells and represent a source for tissue macrophages [36]. Circulating monocytes are able to recognize “danger signals” through specific receptors such as CD14, CD16, CXCR4, and Toll-like receptors [38]. Monocytes can phagocytize and present antigens, secrete chemokines or cytokines, and differentiate in response to infectious lesions and tissue damage [39]. Upon entering tissues, monocytes are able to differentiate into macrophages, dendritic cells, and myeloid-derived suppressor cells [39]. The differentiation of monocytes into macrophages occurs under the influence of monocyte colony-stimulating factor (M-CSF) and interleukin 34 (IL-34), and the functional polarization of macrophages under the influence of interleukin 6 (IL-6), IL-10, TGF-β, prostaglandins, angiotensins, and extracellular vesicles [39,40]. Due to the infiltration into the tumor and the differentiation into TAMs, monocytes can influence the formation of antitumor immunity, angiogenesis, and metastasis [40,41].

The search for new immunotherapeutic approaches, including targeting molecules that determine the degree of antitumor activity of immune cells, is an urgent task of modern oncology. The role of TAMs in the pathogenesis of malignant growth and their proven clinical significance makes TAMs a subject of careful study. There are several approaches, such as blocking monocyte migration into the tissue or destroying TAMs in the tumor, which have reached clinical validation [42,43]. However, none of them have been successfully completed (NCT01015560, NCT02371369). The reprogramming of TAMs by molecules occupying key positions in shaping the functional profile of TAMs represents another attractive strategy for the management of antitumor immunity. The search for molecules whose activity modulation will allow us to reprogram the activity of TAMs or their precursors in order to activate antitumor immunity is currently underway.

Mitogen-activated protein kinase (MAPK) signaling cascades play fundamental roles in many immune response processes and function as master regulators of macrophage pro- and anti-inflammatory cytokine production [18,19,44,45,46]. Three major branches of the MAPK pathway cascade are known, the growth factors extracellular signal-regulated kinase 1 and 2 (ERK1 and ERK2), the stress-sensitive kinases c-Jun N-terminal kinases (JNK) JNK1, JNK2, and JNK3, and the p38 (α, β, δ, and γ) family of kinases [47,48,49]. The production of cytokines, arginase and nitric oxide (NO) production, cell cycle regulation, and control of cell motility are under the control of MAPK. Moreover, the MAPK signaling cascade interacts with molecules of the NF-κB cascade [48]. In general, the ERK1/2 cascade mediates signals that promote cell proliferation, differentiation, or survival, whereas the JNK and p38 MAPK cascades are involved in cellular responses to stress [49]. MAPK signaling cascades are activated in response to a wide range of inducers, including mitogens, hormones, growth factors, cytokines, constituents of oxidative stress, and exposure to physical factors (Figure 1) [50]. The regulation of MAPK signaling cascades in the cell is accomplished by phosphorylation/dephosphorylation in two regions of the conserved TXY domain by dual-specificity phosphatases (DUSPs) [51]. Thus, DUSPs are key molecular regulators of TAMs and their precursors through the control of MAPK signaling cascades.

## 3. DUSPs: Structure and Function

**Figure 2:**
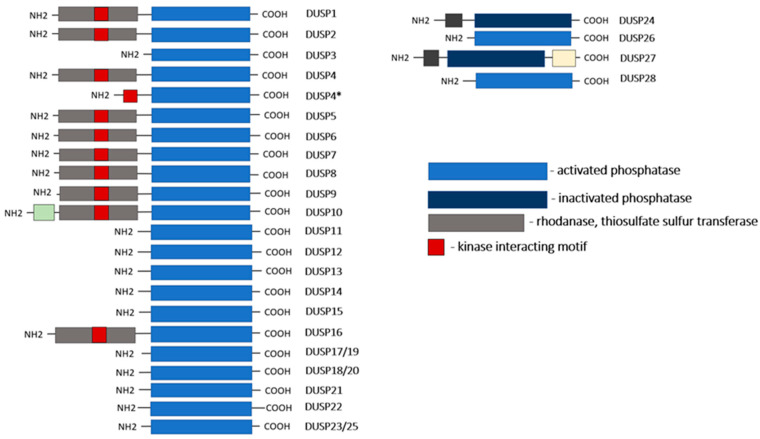
Schematic of DUSP family proteins, which comprises the subfamily of MAP kinase phosphatases (MKPs) and atypical DUSPs. Typical and atypical DUSPs are divided by their structural discrepancy; *—isoform of DUSP4 without rhodanese.

Phosphatases are important controllers of intracellular signaling activities. There are at least six subgroups of dual-specificity phosphatases including the phosphatase and tensin homolog protein phosphatases (PTENs), mitogen-activated protein kinase phosphatases (MKPs), atypical DUSPs, cell division cycle 14 phosphatases (CDC14s), slingshot protein phosphatases (SSHs), and phosphatases of the regenerating liver (PRLs). The co-ordinated interaction of phosphatases and protein kinases in the cell controls the structure, activity, and localization of proteins in the cellular space [16,52,53]. The molecular mechanism of action of phosphatases involves the dephosphorylation of the substrate by hydrolyzing the phosphoric acid ester bond. DUSPs, a family of which currently numbers more than 61 members, 44 of which have been identified in human, have the ability to dephosphorylate both tyrosine and serine/threonine sites [16]. Thus, phosphatases are involved in the regulation of a number of biological events, including the activity of signaling pathways and maintenance of protein stability [15,53,54]. The first DUSP isoform discovered, DUSP1/MKP1, negatively regulates MAPK protein kinase activity by dephosphorylating the TXY motif (Thr-X-Tyr, where X represents any amino acid) in the kinase domain [53] (Figure 2). Although the name DUSPs encompasses a large family of phosphatases, here, we focus on MPK and atypical DUSPs that are important in the regulation of TAMs.

To date, at least 16 DUSP isoforms have been identified in mammals that exhibit dephosphorylation activity toward MAPK (Table 1). Of these, 10 are “typical” MAPK-specific phosphatases (MKPs) that contain a CH2 motif for MAPK docking and comprise three major subfamilies based on their sequence similarity, substrate specificity, and subcellular localization (Figure 2) (Table 1). They all share the common features of having a common phosphatase domain with conserved aspartic acid, cysteine, and arginine residues, thus forming a catalytic site. In addition, their amino acid fragment contains a cluster of basic amino acids as part of the kinase interaction motif (KIM). The KIM confers substrate specificity and is the least homologous region exhibiting an individual substrate preference [55].

Other than “typical” DUSPs against MAPK 6, “atypical” DUSPs also have specificity. These phosphatases, unlike DUSP-MKPs, lack KIM and can have more diverse substrates, including triose-phosphorylated RNA for DUSP11 or phosphatidylinositol phosphates for DUSP23 (Figure 2). Thus, the physiological functions of DUSPs depend on their substrate specificity and phosphatase activity (Table 1). That said, it is quite difficult to characterize the exact substrate domains for DUSPs. For example, the KIM domain approximates MAPK as a substrate for typical DUSPs, but atypical DUSPs without this domain can also efficiently dephosphorylate MAPK [86]. Moreover, even with a relatively conserved KIM, the reported specificities for different MAPKs can vary significantly between typical DUSP members, indicating that DUSP specificity may be refined by regions outside the KIM and phosphatase domains [87].

The regulated expression between cell types and after stimulation, the different compartmentalization of DUSPs in the cell, and the selectivity of binding to MAPK family members determine the specificity of the DUSP action in signal transduction [15]. Given the large number of DUSPs, which are often co-expressed in the same cell type and have overlapping substrate specificity, the redundancy and functional compensation of other phosphatases can be expected when only one DUSP gene is inactivated [15,16,87,88]. However, this redundancy makes it difficult to establish the specific role of a particular DUSP in a particular tissue.

Implying a study of the DUSP activity should take into account that it is an enzyme. Thus, an investigation of the role of DUSPs in macrophage biology can take two directions: the determination of mRNA or protein expression and/or an investigation of the phosphorylation activity of DUSP substrates, MAPK (Table 1). Thus, several isoforms, e.g., DUSP3, DUSP5, DUSP6, DUSP9, DUSP10, and DUSP11, have demonstrated the ability to reduce the phosphorylation of JNK, ERK1/2, p38, and TAK1 [68,70,71,72,76,78,82]. However, no substrate for DUSPs is always known; then, measuring the expression is a possible approach. In this regard, silencing production by genetic knockout, siRNA, or reducing activity by chemical inhibitors may also be methods of choice.

## 4. DUSPs Interaction with Protein Kinases in Macrophage Signaling

On the basis of sequence homology, intracellular localization, and substrate specificity, the family of typical DUSPs has been divided into three groups. The first subfamily includes DUSP1/MPK1, DUSP2, DUSP4/MPK2, and DUSP5. They localize in the nucleus and are triggered by growth factors or stress signals (Table 1). The second subfamily includes three cytoplasmic ERK-specific DUSPs: DUSP6/MKP3, DUSP7, and DUSP9/MKP4. DUSP8, DUSP10/MKP5, and DUSP16/MKP7 constitute the third subgroup, which can be either nuclear or cytoplasmic phosphatases, and they preferentially recognize JNK, p38, or both, respectively [6,7]. Atypical phosphatases are not grouped into common subfamilies. In contrast to “typical” ones, atypical isoforms are involved in the regulation of transcription factors, kinases and phosphatases, and signal transducers, most of which are involved in the regulation of MAPK signaling (Table 1).

**Figure 3:**
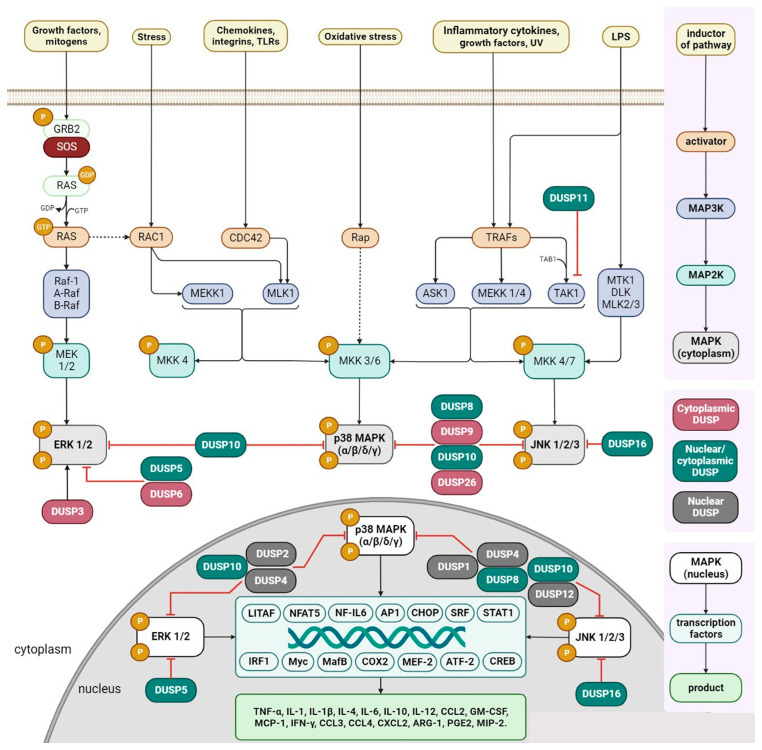
Role of DUPSs in the regulation of the MAPK signaling pathways. The scheme describes the involvement of DUSPs in the regulation of the major MARK signaling ERK 1/2, JNK, and p38 pathways. Mitogens, cytokines, and cellular stresses promote the activation of various MAPK pathways, which, in turn, are dephosphorylated and inhibited by DUSPs.

The first studies of DUSPs in the regulation of the immune response began with the DUSP1 isoform more than 15 years ago [89]. Since then, the regulatory function of other DUSPs for cells of innate and adaptive immunity has been investigated on model cell lines under in vitro conditions, in mouse models, and in human studies. Based on the results of these studies, most DUSP members were found to contribute significantly to the regulation of the immune response and, thus, confirm that MAPK signaling is the primary mechanism for this group of phosphatases [15]. When considering the contribution to the regulation of innate immunity, DUSP1 is the most studied of all DUSPs. It was first detected in macrophages infected with Listeria monocytogenes in 1997 [52]. A 2002 study showed the effect of DUSP1 on the MAPK-kinase pathway in macrophages. Thus, DUSP1 was overexpressed under the influence of LPS, leading to the dephosphorylation of JNK and p38 substrates and the decreased production of the cytokines TNF-α and IL-6 [56] (Figure 3). Although the specific induction of the DUSP family genes and their regulation in macrophages has not been extensively investigated to date, it is known that DUSPs can be overexpressed in immune cells in response to toll-like receptor (TLR) stimulation [15].

Dual-specificity phosphatases belonging to typical DUSPs/MAPK-specific phosphatases and atypical DUSPs are involved in the regulation of macrophage function (Table 1). LPS is the best-known inducer for nuclear MAPK-specific DUSP phosphatases. Under the action of LPS, DUSP1, 2, and 4 inhibit signaling cascades in the p38 and JNK pathways, thereby reducing the production of TNF-α, IL-6, and IL-12 [56,64]. Exposure to Staphylococcus aureus-derived peptidoglycans, zymosan, poly(I:C), and flagellin can also activate DUSP1 in macrophages, leading to the decreased gene expression of JNK, p38, and TNF-α [62,90]. In addition, inducers such as IL-10, together with dexamethasone for DUSP1 or IL-4 for DUSP4, can reduce p38 substrate activity. Meanwhile, dexamethasone is independently capable of inducing DUSP1, leading to the inhibition of the p38 and JNK pathways and the decreased expression of the pro-inflammatory cytokines TNF-α and IL-1 [56,59]. Macrophage colony-stimulating factor (M-CSF) can serve as an inducer for DUSP4, which, in turn, acts to substrate ERK in the MAPK pathway. Interestingly, fatty acids can enhance DUSP4 expression, thereby inhibiting JNK and reducing the ability of macrophages to polarize into the classical phenotype via the JNK/p38 pathway [67]. Physical factors are also inducers of DUSP activity. Thus, heat shock affects DUSP1 by increasing its stability and inhibiting p38 [63].

Mediated by LPS, DUSP5, 8, 10, and 16 inhibit signaling cascades in the ERK and JNK pathways [73,77,80]. Here, DUSP6 is induced under the action of LPS, together with C-C motif ligand 2 (CCL2), leading to the activation of the ERK pathway and a subsequent decrease in the activity of DUSP6 itself [71]. Moreover, M-CSF, as an inducer for DUSP5, reduces ERK substrate activity and alters monocyte differentiation toward granulocytes [69]. In addition to the usual inducers, protein kinase N2 (PKN2) protein kinase is equally capable of inducing DUSP6 in tumor-associated macrophages, reducing ERK activity and the anti-inflammatory cytokines IL-4 and IL-10 [70]. For DUSP8, miR-21 can be considered to be an inducer, which enhances signaling cascades in the p38 and JNK pathways [75]. The inhibition of DUSP10 in macrophages by the siRNA of pharmacological inhibitor AS077234-4 reduces pro-inflammatory cytokine activity and p38 substrate activity [79]. Physical factors such as hyperoxia for DUSP6 enhances ERK substrate activity, and hypoxia/reoxygenation for DUSP9 reduces JNK and p38 substrate activity [71,76].

Atypical phosphatases are mainly induced by LPS. In response to its induction, DUSP12 and 26 inhibit signaling cascades through the p38 and JNK pathways, thereby reducing TNF-α production, and DUSP3 suppresses ERK substrate activity [52,84,85]. In addition, upon LPS activation in macrophages, DUSP11 acts through the substrate transforming growth factor β-activated kinase (TAK1), which is a member of the MAPK kinase (MAPKKKK) family and regulates the NF-κB and MAPK pathways [91]. It is known that TAK1 is able to control the activation or repression of the p38α isoform; thus, there is the possibility of influencing the regulation of the entire p38 pathway through TAK1. Although DUSP7, 14, 18, and 22 can be expressed in myeloid cells, their regulation in macrophages and their effect on the phosphatase activity of MAPK substrates in macrophages have not yet been investigated.

## 5. Regulation of TAM Functions by DUSPs

There are a series of studies that have shown the role of DUSPs in solid tumor progression (Table 2). To date, the relevance of DUSPs in the regulation of macrophages is now revealed, since DUSPs are also expressed in immune cells in the microenvironment, as well as in tumor cells themselves [16,92]. There are different opinions in which cells localizing DUSPs is necessary for tumor development. Nevertheless, it is known that the representation of DUSPs in tumor tissue differs from normal tissue and is associated with the course and prognosis of cancer (Table 2). Some of them can act as tumor growth suppressor genes, while others, on the contrary, support the initiation of tumors. Moreover, each phosphatase, depending on the tumor environment, can gain the properties of an oncogene or an anti-oncogene (Table 2). For example, in breast cancer, DUSP1, DUSP10, and DUSP22 are associated with the stage and prognosis [16,93,94]. Moreover, DUSP4 overexpression or DUSP3 and DUSP2 downregulation correlate with a poor outcome in breast cancer [95,96,97]. For colorectal cancer cohorts, the increased expression of DUSP2, DUSP4, DUSP5, and DUSP22 was a predictor of longer survival rates [98,99,100,101]. In a cohort of prostate cancer patients, decreased DUSP5 levels and DUSP22 overexpression correlate with a worse prognosis [102,103,104]. Ovarian cancer cohorts demonstrated a negative correlation of DUSP1 expression with overall survival, whereas the expression of DUSP2 and gene methylation of DUSP7 and DUSP8 described a positive correlation with overall and recurrence-free survival [16,104,105]. Other solid and blood cancers also show a variable association of DUSP expression with clinical outcomes (Table 2).

Thus, the presence of DUSPs in tumors was demonstrated in studies to correlate with a poor or good outcome. However, these studies are limited to the bulk analysis of tumor tissues and it is difficult to determine whether tumor or immune cells contribute to changes in DUSP expression in the tissue. The accumulation of data on the expression of DUSPs in cell populations using single-cell resolution assays should resolve this difficulty.

To date, the role of MAPK signaling in macrophage polarization and its association with tumor progression is well confirmed [18,44,45,150,151,152]. The activation of the MAPKs can initiate the inflammatory response by the phosphorylation and activation of MAPK-dependent transcription factors, including ETS Transcription Factor ELK1 (ELK-1), c-Jun, activating Transcription Factor 2 (ATF-2), and cAMP Response Element-Binding Protein (CREB), along with the phosphorylation of RNA-binding proteins [153,154]. The upregulation of p38 and ERK1/2 signaling promotes the repolarization of M2-like TAMs toward the M1 subtype, which is accompanied by the increased production of IL-12 and IFN-γ and the suppression of TGF-β release [19,150]. The inhibition of ERK in MAPK signaling suppresses monocyte differentiation into M2 TAMs, which leads to the decreased expression of CD163, IL-10, and the chemokines CCL17 and CCL18 [19,155]. IL-4, which induces the polarization of macrophages into the M2 phenotype, activates JNK signaling and the increased proliferative capacity of cancer cells [155]. In addition, the activation of the JNK pathway under the induction of induced intermittent hypoxia increases the pro-inflammatory phenotype in M0 and M1 macrophages [156]. In general, the activation of MAPK pathway components leads to the differentiation toward pro-inflammatory M1-like macrophages. DUSPs are crucial regulators of MAPK signaling, thereby influencing DUSPs could be one possible therapeutic strategy to targets TAMs.

**Figure 4:**
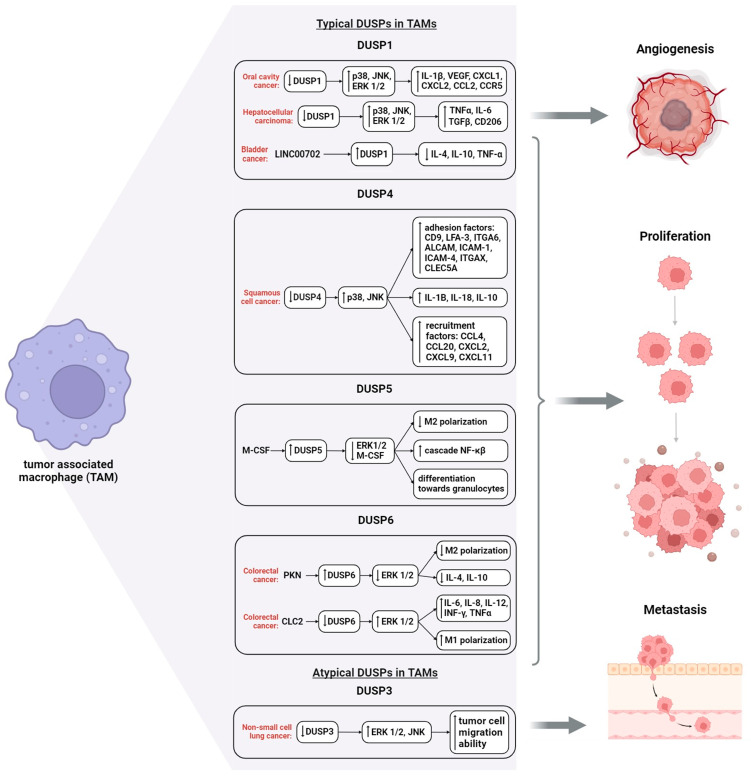
Tumor-associated macrophages’ biological activity related DUSP function. Tumor cells influence macrophage polarization. Depending on the type of carcinoma, the expression level and activity of DUSPs in TAMs change under the influence of tumor cells and their inducers. As a result, TAMs demonstrate properties aimed at support or suppression of tumor growth stages. Note: ↑, increase; ↓, decrease.

Evidence for the ligand- or receptor-specific induction of DUSP family genes and their kinetics in TAMs, as well as the signaling pattern leading to the altered gene expression pattern upon macrophage polarization, is currently limited. Among all DUSPs, the role of DUSP1 in the regulation of TAMs has been investigated to a greater extent [157]. In a hepatocellular carcinoma model, miR-101-reduced DUSP1 macrophages polarized toward M2 were found to have a prolonged activation of ERK1/2, p38, and JNK-stress-activated protein kinase, decreased TGF-β and CD206 expression, and an increased production of TNF-α and IL-6 compared to wild-type cells slowing macrophage-driven hepatocellular carcinoma (Figure 4) [157]. The increased polarization of macrophages toward M2 was also found with a DUSP1 deficiency in an oral cancer mice model, implying that DUSP1 deficiency enhances tumor-associated inflammation [45,158]. Elevated levels of cytokines and chemokines IL-1β, VEGF, CXCL1, CXCL2, C-C motif ligand 2 (CCL2), and CCR5 receptor, and increased leukocyte counts (both histologically and by *Ptprc* gene expression in mice) were observed (Figure 4) [110]. At the same time, it is known that the decreased expression of DUSP1 in oral cancer tissue is due to the hypermethylation of its promoter region [159]. Thus, for hepatocellular carcinoma and oral cancer, the results suggest that the activation of ERK1/2, p38, and JNK may play a crucial role in polarizing TAMs toward the M2 phenotype.

As a result, DUSP1-deficient macrophages exhibited asymmetric polarization profiles: macrophages displayed an enhanced pro-inflammatory phenotype in response to IFN-γ and TNF-α, whereas this deficiency strongly suppressed the M2-like phenotype following IL-4 stimulation. Compared with wild-type mice, DUSP1-deficient mice produced greater amounts of TNF-α, IL-1β, CCL2, granulocyte/macrophage colony-stimulating factor (GM-CSF), IL-6, IL-10, and IL-12p70, which maintained the M2 phenotype in TAMs [110].

The opposite situation is observed in the bladder tumor, where an increase in DUSP1 levels in M2-like OAMs is noted. Bladder tumor cells may affect the polarization of macrophages towards M2, an effect that can be inhibited through the upregulation of DUSP1 by stimulating DUSP1 transcription using the long non-coding RNA LINC00702 [160]. To date known, LINC00702 has already been identified as a tumor-suppressive long non-coding RNA in lung cancer and colorectal cancer [161,162]. Thus, LINC00702-activated DUSP1 suppresses bladder cancer cell proliferation and inhibits the secretion of inflammatory cytokines IL-4, IL-10, and TNF-α M2-OAM in bladder cancer.

Overall, these data support the concept that DUSP1 is a critical negative regulator of the innate immune response. By modulating the activity of both p38 and JNK, as well as ERK, DUSP1 limits both the strength and duration of signals that trigger inflammatory cytokine production.

DUSP3, despite its lack of research, plays an important role as a positive regulator of the innate immune response. The increased expression of DUSP3 has been observed in monocytes and macrophages compared to neutrophils, B cells, or T cells [81]. It was found that DUSP3-deficient macrophages showed LPS-triggered decreased ERK1/2 phosphorylation, whereas the kinetics of p38 and JNK activation were unchanged (studied in sepsis and endotoxic shock). In a model of Lewis lung carcinoma (LLC) metastasis, DUSP3 was a key player in metastatic growth by modulating macrophage recruitment to the lung through various cytokines and chemokines secreted by the carcinoma microenvironment [117]. Associated with the presence of higher monocytes and macrophages, soluble factors secreted by LLC enhance the migration of these cells, by a mechanism involving DUSP3 phosphatase. In the case of LLC metastasis, tumor formation was associated with a higher recruitment of immune cells. It is likely that a single cytokine or a combination of some cytokines secreted by LLC, but not by B16, may contribute to the recruitment of DUSP3^−^/^−^ macrophages, which could explain the differential recruitment of macrophages to the site of LLC metastasis; as the article describes, they compared LLC-luciferase and B16-F10-luciferase melanoma cells administered to mice and saw that there was no difference for B16 in knockout of DUSP3 and its full manifestation, unlike LLC-luciferase.

DUSP4 shows similarities to DUSP1 in its involvement in key molecular cascades: it can modify the innate immune response in a manner similar to that observed in the DUSP1 deletion model through the increased production of IL-6, IL-12, TNF-α, and prostaglandin E2 (PGE2) [15]. In a group of kidney cancer patients, increased levels of DUSP4 mRNA were observed in TAMs compared to blood monocytes [163]. Interestingly, in the same TAMs, the mRNA level of MARK8 was also elevated against the background of the increased gene expression of pro-inflammatory cytokines IL-1B and IL-18 and anti-inflammatory IL-10, although the increased expression of DUSP4 should lead to the opposite (Figure 4). In addition, the TAMs studied in the research showed the activation of the adhesion capacity and the recruitment of monocytes and macrophages into the tissue. The authors explain this heterogeneity of the producing biological inducers by the heterogeneity of the TAM population and recommend the study of the TAM transcriptome by single-cell resolution.

DUSP5 is known as a negative regulator of signal transduction in the ERK1/2 cascade, which leads to the activation of the NF-κB cascade in macrophages [68]. Thereby, the role of DUSP5 in the regulation of inflammatory responses may be more important than that of any other phosphatase, as the NF-κB cascade plays a key role in the regulation of the innate immune inflammatory response in activated macrophages and also affects the expression of many regulatory cytokines, chemokines, receptors, and enzymes such as TNF-α, IL-6, and COX2 [163]. Moreover, DUSP5 is activated by M-CSF in myeloid cells and negatively regulates ERK1/2 activation. Meanwhile, the cytokine M-CSF is known to regulate the production, survival, and function of monocytes and macrophages and activate MARK signaling; M-CSF, especially, regulates the persistent activity of ERK1/2 [69]. Thus, the overexpression of DUSP5 reduces the phosphorylation rate of the ERK1/2 pathway and suppresses M-CSF signaling, leading to a change in progenitor cell differentiation toward granulocytes rather than macrophages [69]. In addition, the inhibition of ERK1/2 in the tumor environment leads to the suppression of macrophage polarization toward the tumor-supporting M2-like direction [69]. One possible mechanism for this process may be the increased expression of DUSP5 through the negative regulation of ERK1/2.

DUSP6 is an important regulator of classical activation in macrophages due to its ability to regulate ERK signaling, thereby modulating cell proliferation, differentiation, and apoptosis [15,71]. In TAM, in colon cancer cells, PKN2-inducible DUSP6 leads to the suppression of tumor-associated M2 macrophage polarization and tumor growth, through the inhibition of the ERK1/2, IL-4, and IL-10 cascade [70]. We also observed a decrease in DUSP6 levels after CCL2 stimulation in bone-marrow-derived macrophages, possibly due to epigenetic changes [71]. Decreased DUSP6 expression enhances classical macrophage activation (in the M1 phenotype), leading to an increased rate of ERK1/2 phosphorylation and the increased expression of pro-inflammatory genes in response to LPS. These results suggest a new role of DUSP6 as a specific negative regulator of classical macrophage activation [71].

Thus, there is currently a paucity of studies on the effects of DUSPs associated with the MAPK signaling pathway. Among all DUSPs, the one most associated with MAPKs is DUSP1; less studied are almost all atypical phosphatases.

## 6. Epigenetic Regulation of DUSPs in Macrophages

Epigenetic control undoubtedly occupies an important place in the regulation of immune cell activity. Such regulation underlies the phenomenon of innate immune memory. The trained phenotype is induced and sustained via epigenetic modifications that reprogram transcriptional patterns and metabolism. Three major mechanisms can be identified for epigenetic regulation—DNA methylation, the regulation of DNA availability via histones, and the blocking of mRNAs by non-coding RNAs such as long non-coding and microRNAs [164,165,166]. The epigenetic level regulation of DUSP expression in macrophages is extremely poorly understood. Therefore, there is a single work of research the about association between the methylation of DUSP genes and DUSP mRNA or the protein presence in macrophages. There is some evidence for the regulation of DUSP expression by histone modifications and non-coding RNAs (Table 3).

**DNA Methylation**

It has been described that the methylation of the DUSP1 promoter is not associated with a decrease in its expression, suggesting the involvement of other factors in the specific repression of DUSP-1 in diabetes-associated cardiac hypertrophy [174]. The knockout or silencing of the main enzyme involved in DNA methylation is provided by DNA Me-thyltransferase 3 Alpha, A.

DNMT3A in macrophages has been shown to suppress the incorporation of methyl groups from AC-methionine into S-adenosyl methionine, SAM, and DNA that reduce the methylation of the Dusp4 promoter and the mRNA expression of DUSP4 [167]. This process leads to the enhancing of ERK signaling and the induction of the PGE2−TGF-β pathway in efferocating (clearing apoptotic cells) CD36+ macrophages.

**Histone Code**

Carried acute viral infections may affect the epigenetic status of DUSP1 in macrophage precursors. Decreased chromatin activity and expression of the DUSP1 gene in clusters of CD14+ monocytes and their precursors were observed in patients who underwent severe COVID-19 [175]. In addition, the activation of DUSP1 gene transcription in murine macrophages by LPS is associated with the acetylation of histones H3 and H4 at the DUSP1 promoter and chromatin remodeling [169]. Interestingly, histone deacetylase isoforms (HDAC1, -2, and -3) deacetylate DUSP1 itself in mice macrophages and this post-translational modification enhances MAPK signaling [176]. The infection of the monocytic THP-1 cell line with H37Rv of M. tuberculosis resulted in the increased methylation of histone H3K4me3 in the promoter regions of the DUSP4 gene and the gene for another immune regulatory protein special AT-rich sequence-binding protein 1 (SATB1). This process was accompanied by the increased expression of lncRNA HOX transcript antisense RNA, HOTAIR, and resulted in the decreased production of the chemokines CXCL1, CXCL2, and CXCL3 in infected THP-1 cells [170].

**miRNA**

A correlation with the DUSPs expression in macrophages has been established for a variety of microRNAs. In RAW264.7 and alveolar macrophages, miR-127 was demonstrated to activate pro-inflammatory activity via targeting genes of B-cell lymphoma 6, BCL6, and DUSP1, remarkably downregulating its expression, and, subsequently, enhanced the activation of JNK kinase [171]. DUSP1 was targeted and downregulated by a mimic of the miR-200 family (miR-429, miR-200b, and miR-200c), followed by the regulation activation of p38 MAPK and the subsequent production of pro-inflammatory cytokines TNFα, IL1β, and IL6 in rat macrophages subjected to LPS treatment [172]. miR-9 mediated the downregulation of DUSP6 and enhanced the ERK-mediated signal transduction, possibly mediating this enhanced pro-inflammatory gene expression in CCL2, and LPS stimulated bone-marrow-derived macrophage mice macrophages (BMDMs) [71]. It is possible to modulate the DUSP-dependent migration capacity of macrophages via microRNAs. Therefore, the knockdown of miR-21 in murine BMDMs directly derepressed the expression of DUSP8, a previously validated miR-21 target in cardiac fibroblasts [75]. These same macrophages demonstrated the impaired chemokine CCL2-induced migration function and impaired macrophage–endothelium interaction activated by TNF-α. In addition, in THP-1 cells, oxidized low-density lipoprotein (Ox-LDL) enhances the upregulation of miR-21 expression, enhancing the phosphorylation of JNK and p38 protein and reducing DUSP8 expression [173]. In this work, THP cells under the induction of Ox-LDL and with elevated miRNA levels demonstrated an increased ability to migrate.

## 7. Targeting DUSPs to TAM Therapeutic Modeling

MAPK protein kinases and the phosphatases that regulate them are responsible for multiple cellular signal transduction processes and the control of cell fates [62,177]. Due to the involvement of DUSPs in the regulation of macrophage activity, their role as targeting molecules is relevant, and the modulation of TAM by DUSPs may be a novel cancer therapeutic strategy. The small size of DUSPs and simple domain structure facilitate the design of molecular inhibitors [178,179]. The advantages of modulating TAMs with DUSPs include targeting phosphosites insensitive to kinase inhibition, as well as combining with kinase inhibitors and/or chemotherapeutic agents to overcome cancer drug resistance [180].

Chemical inhibitors of DUSPs have been found in limited numbers. Earlier this century, (E)-2-(E)-2-benzylidene-3-(cyclohexylamino)-2,3-dihydro-1*H*-inden-1-one (BCI) and its modification BCI-125 were described to block DUSP1 and DUSP6 [181,182]. Regarding triple-negative cancer cells, these agents inhibited their growth and ability to migration [183]. It is currently unknown how BCIs affect tumor-associated macrophages, although BCIs are known to act on macrophages in models of chronic inflammation [184,185,186]. For instance, the pharmacological inhibition of DUSP6 attenuated LPS-induced inflammatory mediators and ROS production in macrophage cells through the inhibition of the NF-κB pathway and the activation of the NF-E2–related factor 2 (Nrf2) signaling axis [184]. BCI suppressed differentiation-related signaling pathways and reduced the differentiation of bone marrow cells into macrophages through the inhibition of DUSP6, which prevented myocardial fibrosis after infarction [185]. BCI reduced DUSP1 levels in infected macrophages and suppressed intracellular mycobacterial proliferation by stimulating cellular ROS production and IL-6 secretion [186].

In addition to BCI, several other inhibitors of DUSPs have been identified. Thus, plant phenanthrene triptolide prevents the LPS-mediated induction of DUSP1 associated with increased levels of p-ERK and p-JNK in model macrophages (Chen et al., 2002). NSC357756 and its analog NU-126 have the ability to inhibit DUSP1, DUSP3, and DUSP6 [54,187,188]. Benzophenanthridine alkaloid, sanguinarine, was able to inhibit DUSP1 and DUSP4 [189,190]. Its administration caused a decrease in the expression of CD206 on IL-4-induced macrophages and inhibited the proliferation, migration, and lumen formation of HUVECs and the expression of CD31 and VEGF [191]. Unfortunately, in this work, the authors did not aim to evaluate the effect of sanguinarine on DUSP and MARK expression in TAMs.

Although sanguinarine, NSC357756, and NU-126 are potent inhibitors of DUSP1, the poor cellular permeability and lack of selectivity are the major hurdles for their therapeutic utilization. The favorable toxicity profile of BCI, its selectivity for DUSP1 and DUSP6, and the ability to inhibit tumor cell proliferation promote further investigation to determine whether this compound targets TAMs and tumor cells. Taken together, these studies suggest that DUSP1 and six antagonists are novel anti-neoplasm compounds for cancer treatment, and further understanding their biological functions will help devise more selective inhibitors with improved cellular permeability.

DUSP1 may play a role as a targeting agent for the therapeutic modeling of TAMs. The non-selective protein kinase inhibitor sorafenib was found to increase DUSP1 expression in the M2 cells of macrophages, resulting in the decreased release of TGF-β and CD206, and, thus, sorafenib better suppresses hepatocellular cancer progression [157]. At the same time, the interruption of DUSP1 in M2 cells may attenuate the antitumor effect of sorafenib. In contrast, the inhibition of one of the phosphatidylinositol-3-kinase (PI3K) signaling pathways important for cancer therapy in murine macrophages resulted in the increased expression of DUSP1 and IL-10, and promoted the anti-inflammatory properties of macrophages [192].

The application of microRNAs is another approach to modulate the properties of macrophages [193]. Mechanistically, miR-127 represses the expression of target B-cell lymphoma 6 and DUSP1, followed by the enhanced activation of c-Jun N-terminal kinase (JNK), which, consequently, promotes the activation of M1 pro-inflammatory macrophages [171]. Similar to miR-127, miR-429 attenuates the translation of DUSP1 and promotes LPS-induced lung injury by boosting the alveolar macrophage production of pro-inflammatory cytokines [172].

It was discovered that gamma irradiation and carbon ion irradiation activate macrophages by the activation of the MAPK signaling pathway, including all three of its complexes: ERK, JNK, and p38. Meanwhile, gamma irradiation at 0.5 Gy increases the expression of DUSP1, which dephosphorylates ERK1/2 and p38 MAPK, leading to the inactivation of p38 MAPK and the decreased production of TNF-α and IL-6 in LPS-activated RAW264.7 macrophages [194]. Since these cells are widely used as a model of the inflammatory response in monocytes/macrophages, similar results would be expected in other macrophage cell lines. Moreover, DUSP1 plays a protective role in human leukemic U937 cells against UV-induced apoptosis by inhibiting UV-induced stress-activated protein kinase (SAPK) activity [88].

Thus, to date, approaches to modulate macrophage function by inducing the expression of DUSPs are in the process of development and selecting a better approach. We should not forget, with regard to DUSP inhibitors, the difficulty and safety of their targeted delivery to the TAM. First of all, DUSP1 and DUSP6 are the targets for modification, but it is possible that other representatives of this family will be selected in the future.

## 8. Conclusions

DUSPs were known for over twenty years to regulate multiple processes included in cell proliferation, migration, and pro- and anti-inflammatory mediator production. DUSPs present in the majority of solid tumors in which the expression of DUSPs correlates with patient survival. DUSPs are key players in tumor cell growth, survival, and death, and have essential roles in tumor initiation, malignant progression, and therapy resistance through the regulation of the MAPK signaling pathway. In parallel, data on the regulation of macrophages via DUSPs were accumulated. The presented review combined two lines of research on DUSPs in cancer. We discussed key points where DUSPs affect TAMs and their precursors, such as the production of cytokines by TAMs, the ability of TAMs and monocytes to carry out adhesion and recruitment to tissue, and the influence on the ability of tumor cells to migrate. Isoforms DUSP1, DUSP4, DUSP5, and DUSP6 are the most promising candidates for TAM modulation. This review provided evidence that DUSPs can be used to reprogram TAMs to activate the antitumor immune response. Thus, we have provided information suggesting a potential for DUSPs to control antitumor immunity, but experimental evidence for this remains to be obtained using current approaches.

## Figures and Tables

**Table 1:** Features of dual-specificity phosphatases as regulators of macrophage function.

Isoforms and Cellular Localization	Inductors	Pathway	Effect of DUSPs in Macrophages
MAPK-specific phosphatases/typical DUSPs			
DUSP1 Nuclear	LPS	p38, JNK	↓ p38 and JNK, ↓ TNF-α, IL-6, IL-10; ↑ IL-12 and IRF-1 (↑ expression of IL-12 by enhancing IRF1 expression) [56,57,58]
	IL-10 + DEX	p38	↓ IL-6, IL-12, p38 (↑ prolonged expression DUSP1) [59,60]
	DEX	JNK > p38	↓ JNK, TNF-α, COX2, IL-1 [56,59]
	Peptidoglycan, Zymosan, poly(I:C), Flagellin	JNK, p38	↓ JNK, p38, TNF-α [61] In DUSP1^−/−^ mF ↑ p38, ↑ TNF-α, IL-10, CD86 and CD40 [62]
	Heat shock	p38	↑ activity of heat shock elements (HSE) in the MKP-1 promoter, stability of MKP-1 mRNA in mF [63]
DUSP2 Nuclear	LPS	ERK, p38 > JNK	In DUSP2 ^−/−^ mF ↓ ERK and p38, Elk1 and NFAT-AP-1 activation ↑ JNK, ↓ TNF-α, IL-6, IL-12α, COX2, IL-1β, C5aR, C3aR + ↓ PGE2, NO [64]
DUSP4 Nuclear	M-CSF	ERK	In DUSP4^−/−^ mF, ↑ ERK ↓ Mmd, Csf2, expression of surface proteins CD115, CD34 [65]
	LPS	JNK, p38	In DUSP4^−/−^ mF ↑ JNK and p38, ↑ ARG-1, IL-6, TNFα, IL-12, PGE2 and ↓ expression of inducible nitric oxide synthase (iNOS) and IL-10, + ↑ DUSP1 as a result of increased ERK signaling [66]
	FFA	JNK, p38	↓ JNK, TNF-α, IL-6, IL-12; ↓ macrophage M1 activation through JNK and p38 [67]
	IL-4	JNK, p38	↑ macrophage M2 activation [67]
DUSP5 Nuclear/cytoplasmic	LPS	ERK	↓ ERK1/2, ↓ ERK1/2 phosphorylation, ↑ NF-κB activity [68]
	M-CSF	ERK	↓ ERK1/2, block macrophage differentiation => differentiate towards to granulocytes [69]
DUSP6 Cytoplasmic	PKN2	ERK	↓ ERK1/2, IL-4, IL-10 [70]
	CCL2+LPS	ERK	↑ ERK1/2 phosphorylation [71]
	Hyperoxia	ERK	↑ ERK [72]
DUSP7 Cytoplasmic	No information		
DUSP8 Nuclear/cytoplasmic	LPS	JNK	↓ JNK, TNF-α, IL-1β, IL-6 [73,74]
	miR-21	JNK, p38	↑ p38, JNK + ↑ macrophage migration and macrophage adhesion to endothelium [75]
DUSP9 Cytoplasmic	hypoxia/reoxygenation	JNK, p38	↓ JNK, p38 ↓ ASK1 phosphorylation, TRAF6, IKKβ (NF^−^ κB pathway), K63 ubiquitination, TNF-α, IL-1β, IL-6 [76]
DUSP10 Nuclear/cytoplasmic	LPS, peptidoglycan, poly(I:C)	JNK [77] + JNK, ERK, p38 [78]	↓ AP-1, TNF-α, IL-6, JNK activity [77] in DUSP10^−/−^ mF ↑ JNK, ERK, p38 phosphorylation, ↑ TNF-α, IL-6, MIP-2, ↑iNOS, ROS production [78]
	Inhibition by siRNA of pharmacological inhibitor AS077234-4	p38	↓ p38, TNF-α и IL-6 [79]
DUSP16 Nuclear/cytoplasmic	LPS	JNK1/2	In DUSP16^−^/^−^ mF ↑ produce IL-12, IRF-1, ↑ JNK 1/2 phosphorylation [80]
Atypical DUSPs			
DUSP3 Cytoplasmic	LPS	ERK	In DUSP3^−/−^ mF ↓ ERK1/2 phosphorylation, Akt, TNF, IL-6 [81]
DUSP11 Nuclear/cytoplasmic	LPS	TAK1	In DUSP^−/−^ mF, ↑ TAK1 phosphorylation (TGF-β–activated kinase 1), TNF-α, IL-6 и IL-1β [82,83]
DUSP12 Nuclear	LPS	JNK, p38	↓ JNK, p38 activation, ↓ expression of AP-1, TNF-α, IL-6, IL-1β, CCL2, ↑ IL-10 [84]
DUSP14 Cytoplasmic	No information		
DUSP18 Nuclear/cytoplasmic/mitochondrial intermembrane space	No information		
DUSP22 Nuclear	No information		
DUSP26 Cytoplasmic	LPS	p38, JNK	↓ TNF-α, p38 significantly and JNK slightly [85]

Note: ↑, increase; ↓, decrease.

**Table 2:** DUSPs’ clinical relevance in cancer.

Isoform	Features in Solid Tumor	Correlation with Cancer Progression Parameters
DUSP1	↑ breast cancer [16,106,107] ↑ prostate cancer [108] ↓ prostate cancer [109], lung cancer by oxidation [106], head and neck squamous cell carcinoma [110]	negative correlation with OS in ovarian cancer [16] positive correlation with DFS in hepatocellular carcinoma [93]
DUSP2	↓ lung, breast, colorectal, prostate, ovarian [111], ↓ ovarian carcinoma [105] ↓ bladder cancer [112] hypermethylation CpG island in head and neck cancer [113]	positive correlation with RFS in Her2+ breast cancer [97] positive correlation with OS and DMFS in colorectal cancer [98] positive correlation with OS in ovarian cancer [105] positive correlation with OS and RFS in bladder cancer [112]
DUSP3	↑ cervical carcinoma, prostate cancer [114,115] ↓ non-small lung cancer [116]	positive correlation with OS for lung, kidney, sarcoma, lymphoma, and breast cancer/negative correlation with OS for lung, breast, and brain cancer for other groups [96] negative correlation with metastasis in non-small lung cancer mice model [117]
DUSP4	↑ medullary thyroid carcinoma, pancreatic cancer, breast cancer, colorectal cancer, rectal cancer, melanoma [99,107,118,119] ↓ serous ovarian carcinoma [120]	negative correlation with OS and DFS in early breast cancer [95] positive correlation with RFS in colorectal cancer [99] positive correlation with OS in lung, pancreatic cancer, gastric, and clear cell renal cell cancer [121] hypermethylation predicts a negative survival factor in B-cell lymphoma [95]
DUSP5	↓ colorectal cancer, prostate cancer, gastric cancer [100,102,122] hypermethylation of CpG islands in gastric cancer [122]	positive correlation with DFS and DSS in colorectal cancer [100] negative correlation with high Gleason score, biochemical recurrence and metastasis in prostate cancer [102] positive correlation with OS in gastric cancer [122] negative correlation with OS in neuroblastoma [123]
DUSP6	↓ melanoma [124] ↓ esophageal cancer [125] ↓ prostate cancer [126] ↓ pancreatic cancer [127] ↑ glioblastoma [128] ↑ thyroid carcinoma [129] hypermethylation of gene promoters in pancreatic cancer [130]	positive correlation with OS, RFS in non-small cell lung cancer [131] positive correlation with OS in esophageal cancer, nasopharyngeal [132]
DUSP7	↓ ovarian cancer [133] ↑ acute and myeloid leukemia [134] hypermethylation of gene promoters in ovarian cancer [104]	methylation-dependent positive correlation with PFS and OS in ovarian cancer [104]
DUSP8	hypermethylation of gene promoters in ovarian cancer [104]	methylation-dependent positive correlation with PFS and OS in ovarian cancer [104]
DUSP9	↓ renal carcinoma [135] ↑ gastric cancer [136] hypomethylation of gene promoters in gastric cancer [136]	positive correlation in renal cancer with OS [135]
DUSP10	↑ ER- breast cancer [94] ↑ prostate cancer [137] ↑ hepatocellular carcinoma, melanoma, lung cancer, colorectal cancer, prostate cancer, glioblastoma [137,138]	
DUSP12	↑ sarcoma, neuroblastoma, retinoblastoma, intracranial ependymoma, chronic myeloid leukemia [139] ↑ DUSP12 positive correlate with c-met and itga, cell migration and genomic instability [138]	
DUSP16	↑ hepatocellular carcinoma [137] ↑ leukemia [140]	
DUSP18	↑ colon cancer [141] ↑ hepatocellular carcinoma [142]	positive correlation with OS in colon cancer [141]
DUSP22	↓ breast cancer [143] ↓ colorectal cancer [101] ↓ peripheral T-cell lymphoma [144] ↓ prostate cancer [103]	positive correlation with OS in IV stage colorectal cancer [101] positive correlation with PFS and DFS in prostate cancer [103] negative correlation OS in anaplastic large cell lymphoma [145]
DUSP26	↓ ovarian cancer, neuroblastoma, medulloblastoma and glioblastoma [146] ↑ thyroid carcinoma [147] ↑ for TGFβ1-promoted EMT in pancreatic, lung cancers cell lines [148]	positive correlation with DFS in neuroblastoma [149]

Note: ↑, increase; ↓, decrease; OS, overall survival; DFS, disease-free survival; RFS, relapse-free survival; DMFS, distant metastasis-free survival; DSS, disease-specific survival; PFS, progression-free survival.

**Table 3:** Features of the epigenetic profile of dual-specificity phosphatase in macrophages and their precursors.

Isoforms	Epigenetic Changes	Key Mechanism	Effects in Macrophages	References
DUSP4	DNA Methylation	Efferocating ↑ DNMT3A ↓ methylation of DUSP4 promoter ↓ DUSP4 mRNA expression	↑ PGE2, TGF-β production	[167]
DUSP1	Histone code	↓ chromatin activity ↑ H3 and H4 histones acetylation ↑ mRNA DUSP1 expression	---	[168]
DUSP1	Histone code	↑ acetylation of histones H3 and H4 at the DUSP1 promoter	---	[169]
DUSP4	Histone code	↑ methylation of H3K4me3 histones	↓ CXCL1, CXCL2, CXCL3	[170]
DUSP1	miRNA	↑ miR-127 ↓ DUSP1 mRNA expression	↑ IL-6, TNF-α, IL-1β ↑ NOS	[171]
DUSP1	miRNA	miR-429, miR-200b, and miR-200c ↓ DUSP1 mRNA expression	↑ IL-6, TNF-α, IL-1β	[172]
DUSP6	miRNA	↑ miR-9 ↓ DUSP6 mRNA expression	↓ NOS (after 6 h of CCL2 induction) ↑ TNF-α	[71]
DUSP8	miRNA	↓ miR-21 ↑ DUSP8 mRNA expression	↓ CCL2-induced migration ↓ macrophage-endothelium interaction	[16]
DUSP8	miRNA	↑ Ox-LDL ↑ miR-21 ↑ DUSP8 mRNA expression	↑ migration	[173]

Note: ↑, increase of expression or activity; ↓, decrease of expression or activity; DNMT3A—DNA methyltransferase 3 alpha; PGE2—Prostaglandin E2; TGF-β- transforming growth factor-β; H3K4me3—trimethylation of histone H3 lysine 4; CXCL1, 2,3—chemokine (C-X-C motif) ligand 1,2,3; IL-6—interleukin-6; TNF-α—tumor necrosis factor-alpha; IL-1β—interleukin-1β; NOS—nitric oxide synthase; Ox-LDL—oxidized low-density lipoprotein; CCL2—C-C motif ligand 2.

## Data Availability

Not applicable.

## Abbreviations

Akt	aktine
ALCAM	transmembrane glycoprotein CD166
AP1	activator protein 1
ARG1	arginase
ASK1	apoptosis signal-regulating kinase 1
ARG1	arginase 1
ATF2	activating transcription factor
C3aR, C5aR	complement component 3a, 5a receptor 1
CD9	tetraspanin family protein
CHOP	C/EBP homologous protein
CLEC5A	C-type lectin domain family 5 member A
COX2	COX-2 inhibitors
CREB	cAMP response elements beta
c-Src	Src cytoplasmic proto-oncogene-tyrosine-protein kinase
CXCL	chemokine (C-X-C motif) ligand
DLK	double leucine zipper kinase; MKK—MAPK kinases
DUSP	dual-specificity phosphatases
EGF	epidermal growth factor
EGFR	epidermal growth factor receptor
Elk1	ETS Like-1 protein
ERK	extracellular signal-regulated kinase
FADD	Fas receptor death domain-interacting protein; MEKK, MEK kinase
FAK	focal adhesion kinase (proteintyrosine kinase)
FasL	ligand for Fas membrane molecule
FasR	receptor for Fas membrane molecule
FFA	saturated free fatty acid
GRB2	protein with SH2 and SH3 domains
ICAM	intercellular adhesion molecule 1
IGF1R	insulin-like growth factor receptor 1
INFγ	interferon gamma
IRF1	interferon regulatory factor 1
ITGA6	integrin alpha 6
ITGAX	integrin, alpha X, CD11c
JNK	c-Jun N-terminal kinase
JNK	c-Jun N-terminal kinase; MAPK, mitogen-activated protein kinase
LITAF	lipopolysaccharide Induced TNF Factor
LFA3	lymphocyte function-associated antigen 3
LPS	lipopolysaccharides; GR, growth factors
MEK	MAPK/ERK kinase
MEF2	myocyte Enhancer Factor 2
mF	macrophages
MIP2	major Intrinsic Protein 2
MKP	mitogen-activated protein kinase phosphatase
MLK3	mixed origin kinase 3, serine/threonine protein kinase
Mmd	monocyte to macrophage differentiation associated
MSK	mitogen and stress-activated protein kinases
NFAT	nuclear factor of activated T-cells
NO	nitric oxide
PGE2	prostaglandin E2
PKN2	protein kinase N2
Raf	protein belonging to the serine-threonine protein kinase family;
RAS	inactive membrane-bound small G protein; RAS-GTP, active RAS protein
ROS	reactive oxygen species
SOS	guanine nucleotide substitution factor
STAT	family of proteins that are intracellular transcription factors
SRF	serum Response Factor
TAB1	mitogen-activated protein kinase, regulator of MAP3K7/TAK1 kinase kinase
TAK1	transforming growth factor β-activated kinase 1, a member of the MAPK family
TGF-β	transforming growth factor beta
TNF-α	tumor necrosis factor α
TRAF6	TNF receptor associated factor
TrkA	tyrosine kinase A
UV	ultraviolet radiation
VEGF	vascular endothelial growth factor
